# Histopathologic Concerns and Diagnostic Challenges in Hirschsprung’s Disease: An Eastern European Single-Center Observational Study

**DOI:** 10.3390/life15030329

**Published:** 2025-02-20

**Authors:** Emőke Horváth, Zoltán Derzsi, Eliza Löckli, Gyopár-Beáta Molnár, Zsolt Bara, Evelyn Kovács, Horea Gozar

**Affiliations:** 1 Department of Pathology, George Emil Palade University of Medicine, Pharmacy, Science and Technology of Targu Mures, 540142 Targu Mures, Romania; emoke.horvath@umfst.ro; 2 Pathology Service, Targu Mures, County Emergency Clinical Hospital, 540136 Targu Mures, Romania; gyopar.molnar@umfst.ro; 3 Department of Pediatric Surgery and Orthopedics, George Emil Palade University of Medicine, Pharmacy, Science and Technology of Targu Mures, 540142 Targu Mures, Romania; zsolt.bara@umfst.ro (Z.B.); horea.gozar@umfst.ro (H.G.); 4 Clinic of Pediatric Surgery and Orthopedics, Targu Mures, County Emergency Clinical Hospital, 540136 Targu Mures, Romania; evelynkovacs@yahoo.com; 5 George Emil Palade University of Medicine, Pharmacy, Science and Technology of Targu Mures, 540142 Targu Mures, Romania; elizalockli19@gmail.com; 6 Department of Biochemistry and Environmental Chemistry, George Emil Palade University of Medicine, Pharmacy, Science and Technology of Targu Mures, 540142 Targu Mures, Romania

**Keywords:** aganglionosis, calretinin, CD56 and S-100 immunohistochemistry, nerve trunk hypertrophy, diagnostic algorithm in rectal biopsies

## Abstract

Background: We proposed a comprehensive clinicopathological study involving the characterization of the study cohort and a comparative analysis of biopsies and surgical specimens from patients with Hirschsprung’s disease. The study was complemented by the diagnostic value of calretinin, CD56, and S-100 immunohistochemistry. Methods: Descriptive statistical analysis of diagnostic variables in the group of biopsy specimens (n = 32) and bowel resection specimens (n = 16) was performed. The pattern of calretinin and CD56 expression in Meissner’s plexus elements was analyzed and the maximum thicknesses of the nerve fibers were measured using morphometry with S100-immunostained sections. Conclusions: Coupled calretinin–CD56 immunohistochemistry is useful in diagnosing ganglion cell paucity biopsies or specimens with incomplete submucosa. In cases where there are no ganglion cells but there are calretinin-positive nerve fibrils in the lamina propria without nerve trunk (NT) hypertrophy, re-biopsy is the best solution. The significant differences in NT size between biopsies and surgical specimens highlight the importance of assessing NT diameter in all tissue samples examined.

## 1. Introduction

Intestinal peristalsis is coordinated by the enteric nervous system (ENS), which is derived from neural crest cells (NCCs) that populate the intestinal wall by migrating along the intestine; then, they proliferate and differentiate into enteric neurons and glia. These complex mechanisms are under the molecular control of numerous signaling pathways, transcription and neurotrophic factors, and extracellular matrix components. The dysregulation of these processes leads to enteric neuropathies [1]. The best known of these is undoubtedly congenital Hirschsprung’s disease (HD) or aganglionic megacolon, characterized by a variable degree of contiguous submucosal and myenteric aganglionosis, extending proximally from the anorectum (dentate line). The rectosigmoid form (RsHD) is the most common type (75–80%) [2]. Long-segment colonic Hirschsprung disease (LSCHD; 10–15%) [3] and total colonic aganglionosis with ileal involvement (TCa; 5–7%) are rare, and the latter has an unfavorable prognosis [4,5]. Due to variability in clinical signs, the diagnosis or exclusion of Hirschsprung’s disease (HD), especially in short-segment forms, is a daily challenge in every pediatric and pathology department [6]. If the clinical picture and the subsequent imaging findings suggest a likelihood of HD, then a biopsy is mandatory [7,8,9].

Histopathological confirmation is the gold standard of diagnosis, with an accuracy of 95%; it can reach a sensitivity of 99.7% with immunohistochemical reactions. This examination includes the processing of biopsies from two investigative steps to map the ganglionic–aganglionic junction and the most distal level of normal innervation: (a) rectal biopsy to detect aganglionosis in all forms of the disease (2 and 5 cm from the dentate line); and (b) proximal bowel biopsies or “leveling”. The location of leveling biopsies is based on a clinicoradiologic suspicion of the extent of involvement [10].

The diagnosis of HD requires representative tissue samples [7]. There are several forms of biopsy sampling, the most common being rectal suction biopsy (where only the mucosa and submucosa are present), as well as punch biopsy and open biopsy [11], which are conducted along the posterior wall of the rectum at a distance exceeding 2 cm from the dentate line to circumvent the physiologically hypoganglionic anorectal region [12]. It is not advisable to perform anterior biopsies due to the potential risk of perforation into the vaginal wall or peritoneal cavity [8]. For this anatomical region, the technique, patient positioning, and anesthesia are also more complicated. In the event that an adequate number of high-quality samples are available, and the biopsy contains an appropriate proportion of mucosa and submucosa (minimum 3:1 ratio), a pathologist with experience in this field can often make a definitive diagnosis of HD based on conventional hematoxylin and eosin (H&E)-stained sections. The existence of a single ganglion cell makes the possibility of HD implausible if this condition is met [8]. Nevertheless, this remains a significant challenge, particularly when examining rectal biopsies obtained through aspiration. These biopsies often contain incomplete submucosa, which makes it challenging to identify ganglion cells, which are individually placed and randomly distributed [13].

Additional diagnostic tests, such as acetylcholinesterase histochemistry (AChE) and immunohistochemistry (IH), are invaluable in supplementing the histopathological diagnosis of HD [14,15,16]. The histochemical diagnosis of HD is based on the observation that the cholinergic nerve fibers of the aganglionic segment contain an increased amount of AChE. Consequently, the extent of AChE-positive nerve fiber growth is likely to correlate with the clinical severity of HD [17]. As a consequence of the creation of human-made products and the technical challenges associated with flash freezing, a number of research institutions have begun to develop expertise in immunohistochemical staining using antibodies (calretinin, S-100, NSE, CD56) that effectively visualize ganglion cells and their surrounding supporting cells on paraffin-embedded sections [18,19]. An algorithm is of particular importance when analyzing biopsies from newborns, as immature ganglion cells can be mistaken for endothelial cells or fibroblasts [20]. In cases where children with clinical signs of HD and in poor general condition are to undergo colostomy, the opinion of the pathologist is crucial. This can confirm the presence of ganglion cells in the bowel segment proximal to the colostomy site or ileostomy in total colonic aganglionosis [13]. During surgery, the pathologist plays a crucial role in identifying the ganglionic area and then excluding transitional zone features (partial circumferential aganglionosis, myenteric hypoganglionosis, and submucosal nerve hypertrophy) at the stomy border. The histological analysis of the resection specimens provides detailed information regarding the length of the aganglionic segment and its proximity to the stomy edge, thereby confirming that the transition zone has been completely resected [21].

The present clinical–pathological study aims to correlate and compare biopsies with surgical specimens from patients with HD and proposes to establish a simple algorithm and a minimal panel of immunohistochemistry antibodies to be used in the diagnosis of HD.

## 2. Materials and Methods

### 2.1. Patients and Tissue Fragments

A retrospective analysis was performed of cases submitted to the pathology service of Targu Mures County Emergency Hospital (Romania) with a clinical diagnosis of HD between 2016 and 2023. A total of 48 cases with a histopathological diagnosis of HD were selected for clinical–pathological study according to strict criteria. Cases were selected from pediatric patients meeting the following criteria: aged ≤ 18 years; complete clinical documentation; biopsies taken at least 2 cm above the dentate line; biopsies of sufficient quantity and quality for histological evaluation; and biopsies with a minimum diameter of 0.2 cm and showing a significant amount of submucosa in addition to mucosa (according to recommendations) [22]. Personal and clinical data, including age, sex, comorbidities, complications, and the number of admissions, as well as diagnostic variables (such as the number of biopsies and their distance from the dentate line, biopsy quality, the presence or absence of complete or incomplete submucosa, the width of the nerve trunks (NTs), the extent of aganglionosis, the follow-up period, and the outcome), were recorded and processed for the biopsy group (n = 32) and the bowel resection group (n = 16). The control group was represented by 16 biopsies from patients with suspected HD but for whom a positive diagnosis was excluded by normal rectal tissue structure.

This study was conducted in accordance with the Declaration of Helsinki. The Medical Ethics Committee of Targu Mures County Emergency Hospital (No. 29529/13.11/2023) gave its approval for this study to proceed. Informed consent was signed by the close adult relatives of all patients.

### 2.2. Histopathology and Immunohistochemistry

**Figure 1:**
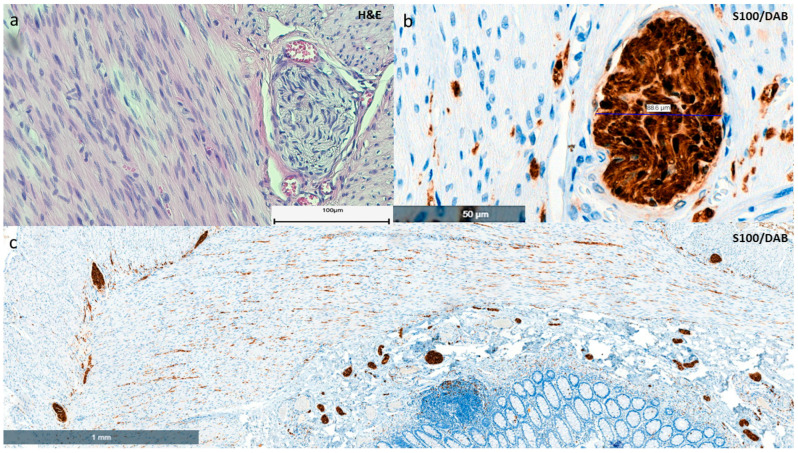
(**a**–**c**) A histological section of a resected colon–aganglionic segment from a patient with HD. (**a**) Myenteric aganglionosis associated with nerve trunk hypertrophy (H&E staining; scale bar 100 µm). (**b**) The exact diameter of the nerve trunk was determined on S-100-immunostained sections using digital morphometry (×400 magnification). (**c**) Myenteric and submucosal nerve hypertrophy in aganglionosis (rectum defined as ≥40 µm in diameter) is considered to be supportive of the diagnosis of HD. Active mucosal inflammation (enterocolitis) may also be observed.

All fragments were processed according to standard methodology. The morphological characteristics of incomplete intestinal wall structures (biopsy specimens) were examined in 4–5 µm sections stained with hematoxylin and eosin (H&E). Immunohistochemistry was performed using an automated system to highlight ganglion cells, intrinsic nerve fibers (INFs), and nerve trunks (NTs). The immunohistochemical panel consists of three complementary antibodies: anti-calretinin (clone: DAK-Calret1, Dako, Santa Clara, CA, USA), anti-CD56 (clone: 123C3.D5, SCBT, Dallas, Texas USA), and anti-S-100 (Z0311, Dako, Agilent, Santa Clara, CA, USA). These were used in combination with EnVision FLEX/HRP (Agilent, Dako, Santa Clara, CA, USA) as secondary antibody and 3,3’-diaminobenzidine chromogen (DAB), respectively, according to the manufacturer’s instructions. The 32 biopsy specimens were divided into two categories based on architectural appearance: complete submucosa (thickness of submucosa approximately equal to that of mucosa) and incomplete submucosa. Incomplete submucosa was considered if the submucosa was less than one-third of the total biopsy [8]. Biopsies without ganglion cells but with non-hypertrophied nerve fibers were re-sectioned to obtain at least ten multi-layer sections, which were then supplemented with calretinin and CD56 immunostaining. Immunostaining with these antibodies was performed to detect residual ganglion cells and associated intrinsic nerve fibers (INFs) that may have been masked by processing artifacts. S100 staining was used to visualize and measure the diameter of the NT on both the study biopsies and the control biopsies. Any false-positive or false-negative results were excluded based on the positive endogenous control for each antigen tested. This included mast cells for calretinin, NK cells for CD56, and nerve fibers or adipocytes for S-100. In cases where the submucosa was incomplete but the calretinin immunolabeling of INFs in the lamina propria and muscularis mucosae was positive, serial sections were extended until the material was exhausted, thus increasing the likelihood of identifying the presence of any ganglion cells. The expression patterns of calretinin and CD56 in biopsies (multi-layer sections) were analyzed and compared between specimens with complete and incomplete submucosa. As the absence of ganglion cells is typically associated with hypertrophy of the NFs, we also analyzed the thickness of the NFs in both the biopsy and surgical specimens. The maximum thickness of the NFs was measured by morphometric methods on S100-immunostained sections for each individual case after the digitization of these sections, using a cut-off value of ≥40 μm as a reference value for nerve enlargement [23,24]. The extent of aganglionosis (short segment, long segment, total form) was determined through the comprehensive analysis of bowel resection specimens, including their serial biopsies. All cases in which aganglionosis was observed exclusively in the rectosigmoid and descendent colon were classified as short-segment cases. If aganglionosis was found to extend beyond the splenic flexure into the transverse colon, it was classified as long-segment aganglionosis. Cases in which ganglion cell loss involved the entire colon or extended proximally into varying lengths of the small bowel were considered to be total, and “extensive aganglionosis” was defined as cases in which the aganglionic segment extended into the jejunum [25]. Specimens obtained during colostomy closure and final lowering were used to assess the proximal end for the presence of ganglion cells. The identification of a single ganglion cell in biopsy specimens was considered sufficient to rule out the possibility of aganglionosis [8]. As the absence of ganglion cells is typically associated with nerve fiber hypertrophy, we also examined the thickness of nerve fibers in the biopsy samples. Maximum nerve fiber thickness was measured by morphometric methods on S100-immunostained sections for each individual case after the digitization of these sections using a Histech 3D digital scanner (Figure 1). A cut-off value of ≥40 μm was used as a reference for NT enlargement to determine the maximum diameter of the NTs [23,24].

### 2.3. Statistical Analysis

Continuous variables are expressed as mean ± SE. A comparison of 2 × 2 distributions was performed using Fisher’s exact test, while the Mann–Whitney U test was used for continuous variables. A *p*-value of 0.05 was generally considered to be statistically significant.

## 3. Results

### 3.1. Clinical Data

The total population consisted of individuals aged 0–16 years, with the majority of patients in the 1–3-year-old age group. Only 22.91% of patients were less than one year old, with a relatively even distribution between patients aged one–three years (39.58%) and over three years (37.5%). The gender distribution showed that the majority of patients (70.83%) were male (34 cases). A relatively high number of patients (six) had a diagnosis as a result of emergency surgery for complications of the disease (including Hirschsprung-associated enterocolitis, obstruction, or bowel perforation). Of the 46 patients diagnosed with HD, 9 had significant comorbidities, including isolated anomalies such as cardiac defects (2 cases), gastrointestinal malformations (like intestinal malrotation), inguinal hernia, genital anomalies (phimosis and hypospadias), genetic syndromes (Down syndrome), and neuropsychiatric disorders (epilepsy and behavioral disorders).

### 3.2. Biopsy Specimen Group Characteristics

#### 3.2.1. Patient Data and Biopsy Techniques

During the study period, 32 patients with clinical symptoms (delayed passage of meconium, abdominal distension, bilious vomiting, and severe constipation or encopresis) and imaging features suggestive of Hirschsprung’s disease underwent suction rectal biopsy or open biopsy according to established guidelines, depending on the patient’s age [26,27,28]. In general, suction biopsies were used in children under six months of age, while open biopsies were mainly used in older children or those with a history of inconclusive biopsies.

A total of 66 biopsies were analyzed. All rectal biopsies were taken from the posterior wall approximately 2–3 cm above the dentate line (mean 3.1 ± 0.7 cm) to avoid the physiological hypoganglionic zone. In the majority of cases, two rectal biopsies were taken. However, in nine cases, biopsies were taken from different levels of the colon. Almost all patients had only one intervention, with the exception of one 12-year-old patient. He had a repeat biopsy a relatively long time after the first attempt, at 15 years and 6 months. Of the 32 patients with HD, 19 (59.4%) were male. The age of the subjects ranged from 15 days to 15 years. A total of eleven patients (34.38%) were diagnosed before the age of one year, including eight males and three females. Only five patients (15.63%) were diagnosed in the neonatal period, all of whom were male.

#### 3.2.2. Histopathology and Immunohistochemistry Analysis

The diagnosis of rectal aganglionosis or colic aganglionosis was based on the absence of Meissner’s plexus ganglion cells associated with hypertrophy of the NTs, and it was diagnosed in only seven cases on the basis of a single biopsy Figure S1; in the majority of cases, at least two biopsies were taken (78.2%). In total, a suboptimal amount of submucosa was identified in five cases (15.625%). Despite the use of serial sectioning until material was exhausted and the performance of calretinin IHC on both initial and final sections, the presence of ganglion cells in these biopsy fragments could not be confirmed. However, in three cases, calretinin-positive nerve fibers were found in the lamina propria and muscularis mucosa. These cases (9.38%) were considered inconclusive, and it was recommended that the biopsy be repeated to avoid a false-positive diagnosis of aganglionosis. With regard to positive CD56 immunolabeling, we observed a discrepancy in the overlap with calretinin expression, with the number of cases showing double-negative labeling being significantly higher (*p* = 0.012) in comparison with cases showing calretinin-negative but CD56-positive cells. In the absence of calretinin immunolabeling associated with NT hypertrophy, we postulated that these labels were immunostaining for glial cells.

The mean NT thickness was 42.2 ± 2 µm, with a range of 20 to 55 µm. The hypertrophy of the NT associated with aganglionosis was significantly higher than that of those with a normal diameter (*p* < 0.001). None of the control group biopsy fragments showed NT hypertrophy. We note that the cases that presented with this malformation alone represented a significantly higher percentage (*p* = 0.022) than those associated with other comorbidities.

The descriptive statistics for diagnostic variables in the biopsy specimen group (n = 32) are shown in Table 1.

### 3.3. Surgery Undergone and Sample Group Characteristics

This group includes patients (16) who were clinically diagnosed with HD in association with severe enterocolitis, toxic colon syndrome, or obstruction, and who required emergency bowel resection. At the same time, multilevel biopsy sampling was performed to evaluate the aganglionic bowel segment. Of 16 surgically treated patients, 10 had a previous diagnosis based on rectal biopsy histology (7 with confirmed aganglionosis and 3 with inconclusive histopathological findings with recommendation for re-biopsy). The remaining patients had no previous hospitalizations in our medical records. The remaining 22 cases were not admitted to pediatric surgery during the study period.

The mean postoperative follow-up period was 3.8 ± 0.5 years, ranging from 1 to 12 years. These patients who underwent surgery both pre- and postoperatively had relatively more hospital admissions (mean = 6.067) in both the surgical and the pediatric unit, predominantly for lower respiratory tract infections.

The data for this group are summarized in Table 2, before a more detailed analysis is provided.

Of the patients, 81.2% were admitted in an emergency for the management of bowel obstruction. Among our results, the relatively high percentage of cases (31.25%) that were resolved in two steps, using temporary ileostomy or colostomy, should be highlighted. Short-term postoperative complications after surgery were significantly rare (25%), such as lower gastrointestinal bleeding, clostridium enterocolitis, and wound dehiscence, compared to long-term outcomes (75%), the most important of which were fecal incontinence, soiling, obstructive syndromes, and adherence syndrome.

Table 3 shows the correlation between the type of HD and the number of hospitalizations from the onset of symptoms to the end of the follow-up period, as well as the correlation between HD and other associated malformations. The results presented indicate a positive association between the number of hospitalizations and long-term complications and the degree of aganglionosis. The association with other malformations does not seem to depend on the specific type of aganglionosis, but interpretation is limited by the small number of cases with double malformation, which included intestinal malrotation, hypospadias, and two interatrial septal defects.

**Figure 2:**
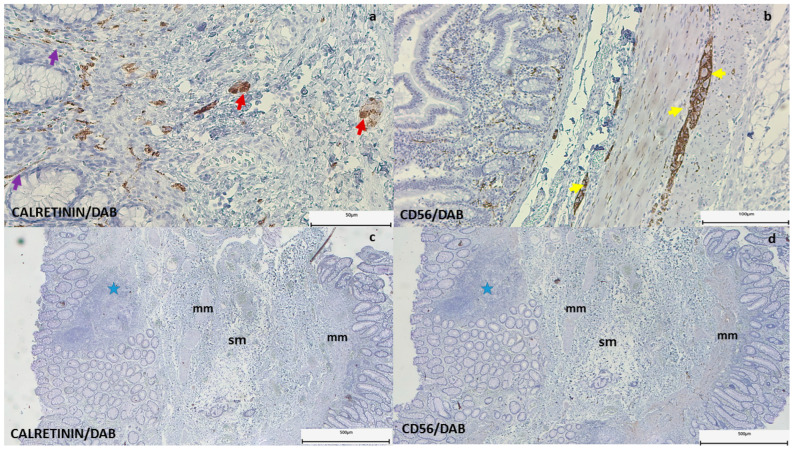
(**a**–**d**) Calretinin and CD56 immunohistochemistry in patients diagnosed with Hirschsprung’s disease. (**a**) A histological section of a resected colon: normal segment from a patient with HD showing calretinin/DAB-positive ganglion cells in the submucosa (red arrows) and their extended nerve fibers in the lamina propria (purple arrows). Scale bar: 50 µm. (**b**) CD56/DAB staining visualizes the ganglion cells of the muscularis propria and submucosa (yellow arrows). Scale bar: 100 µm. (**b**) CD56/DAB-positive ganglion cells of the muscularis propria and submucosa (yellow arrows). Scale bar: 100 µm. (**c**,**d**) Rectal biopsy of the patient showing disorganization of the muscularis mucosae and lymphocytic infiltrate in the mucosa (blue star), but with negative staining for calretinin (**c**) and CD56 immunohistochemistry (**d**). Scale bar: 500 µm. Note: m—mucosa; mm—muscularis mucosae; sm—submucosa.

The histopathological examination of surgical specimens unanimously confirmed the absence of ganglion cells. Immunohistochemistry for calretinin and CD56 was used to analyze the stacked biopsies taken during the colostomy–ileostomy procedure or to characterize the resection margins, which were complete in 87.5% of cases (14), or to characterize the extension of the aganglionic segment (Figure 2). In terms of type, the long segment predominated (68.75%). However, we also identified a total form.

In two cases (12.5%), rare dysplastic ganglion cells were present at the proximal resection margin, immunolabeled only with CD56 and associated with nerve fibers not exceeding 40 µm.

At the level of the aganglionic segment, hypertrophy of the nerve fibers was observed in all cases, with a diameter of 87.6 ± 1.2 (mean ± SE).

### 3.4. Comparative Analysis of Clinical and Histopathologic Findings in the Two Study Groups

The results of a comparison between the variables monitored during the histopathological examination of the biopsy specimens and those recorded during surgery are presented in Table 4.

The most significant results are shown in Table 4, of which only nerve fiber thickening is statistically relevant, highlighting the importance of evaluation together with ganglion cells on serial sections of biopsies to avoid false-negative diagnoses. In two cases, clusters of CD56-positive, calretinin-negative cells were also found on some sections of the aganglionic segment. Compared to the ganglion cells present at the proximal resection margin, these cells were smaller and had nuclei with inconspicuous nucleoli.

### 3.5. Diagnostic Algorithm of Hirschsprung’s Disease in Rectal Biopsies

**Figure 3:**
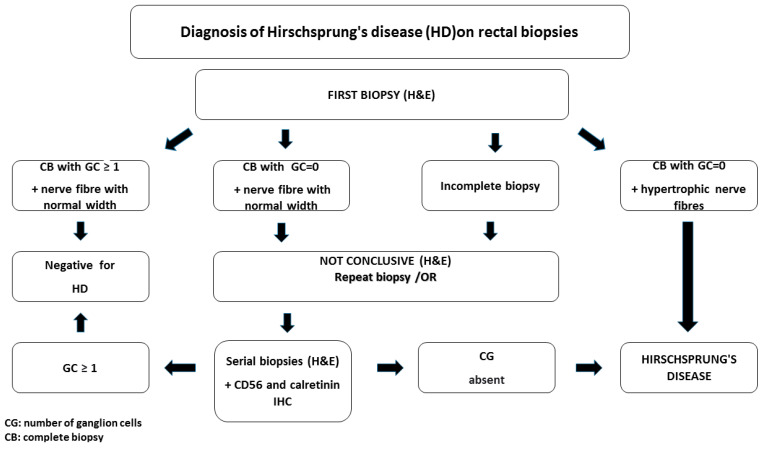
Algorithm for the diagnosis of Hirschsprung’s disease in rectal biopsies.

On H&E-stained sections, the direct signs (absence of ganglion cells associated with nerve fiber hypertrophy, penetration of thickened fibers into the mucosal muscle) are evident in biopsies with a preserved mucosa/submucosa ratio. However, the method has limitations, especially in the case of suction biopsies in premature infants with immature ganglion cells or incomplete biopsies. In these cases, serial sectioning and calretinin immunohistochemistry is the main method for identifying GCs and associated intrinsic nerve fibers of the muscularis mucosae and lamina propria. The absence of ganglion cells associated with hypertrophied nerve trunks and calretinin-positive nerve fibrils in the lamina propria, muscularis mucosae, and superficial submucosa should be interpreted with some caution. In these cases, re-biopsy is the optimal solution. Figure 3 shows the sequence of histopathological examination of HD on paraffin-embedded biopsies used by our group.

## 4. Discussion

HD is the most common congenital intestinal motility disorder; it can lead to life-threatening complications due to functional bowel obstruction, and is increasing in prevalence, slowly but significantly, in Europe [29]. Regardless of geographical location, the disease is more common in males and isolated cases are more common than those associated with other malformations [30]. Associated congenital anomalies vary in different studies, but when all forms of anomalies are considered, the prevalence reaches 18% [31,32]; in this context, HD can be considered a multifactorial malformation linked to many genetic variants and genes [30,33]. Despite the relatively modest size of our study group, the relatively high prevalence of additional associated congenital malformations underlines the complexity of the disease.

In terms of personal data, the gender distribution of HD in our cases showed a clear male predominance, consistent with the literature data [34,35]. On the other hand, the mean age was significantly higher in our population, both at the time of biopsy and at the time of surgery. This discrepancy with data in the literature applies to all age groups [9,36]. The shift toward later infancy with severe and chronic constipation but without major complications suggests that the main signs of the disease are underestimated by both parents and pediatricians, being attributed to common milestones (weaning, toilet training, start of pre-school education) or other life events. If these age-related causes are excluded and a balanced diet and adequate fluid intake are maintained, the presence of infrequent bowel movements (≤2 per week), fecal incontinence, fecal retention, painful or hard stools, or a large stool diameter suggests functional constipation, which may be caused by HD, hypothyroidism, hypercalcemia, spina bifida/spina bifida occulta, and medications that slow bowel motility [37]. In our case reports, 81.2% of patients with aganglionic segment removal were admitted in an emergency for the management of complications, and a relatively high percentage of cases (31.25%) required a two-stage solution with temporary colostomy–ileostomy. Despite short-term postoperative complications, long-term outcomes were present in 75% of cases, the most important of which were soiling, obstructive syndromes, and adherence syndrome, which were also reported as the most common complications of extended procedures [38]. In this context, early diagnosis is important for avoiding major complications (e.g., intestinal obstruction, enterocolitis, or toxic megacolon) that negatively affect treatment outcomes.

Over the past 20 years, rectal biopsy has been recognized as an important diagnostic tool, providing a definitive diagnosis of HD with an accuracy of 95% [10]. This implies the advantage of histologically confirmed aganglionosis and nerve trunk hypertrophy in rectal biopsies; it is known that biopsies of rectal submucosa every 20 μm have a very high probability of being useful for finding a ganglion plexus in the bowel with normal innervation [39].

Although most studies emphasize that the evaluation of GCs in rectal biopsies is not a challenge for an experienced pathologist, daily practice contradicts this statement for several reasons; the most important of these reasons is related to the variable quality of biopsies. This variability is due to the heterogeneity of rectal biopsy techniques, the age of the patients, their frequent comorbidities, indications for the number of biopsies, or the different, often controversial histological/immunohistochemical processing method that is used to make a definitive diagnosis. The number of biopsies performed in our case series is consistent with those recommended in the literature [40], bringing our inadequate sample rate within the acceptable range.

It is generally accepted that the presence of a single ganglion cell in the myenteric plexus or submucosal plexus on H&E sections can exclude the diagnosis of HD. There are suggestions in the literature that at least 50 sections should be examined to confirm aganglionosis, while others suggest that 10 sections from different levels of the specimen are usually sufficient to detect the presence of ganglion cells [41]. In some studies, the authors state that the number of biopsies should be at least two [42], and their diameter should be at least 3 mm [43]. It is sufficient if at least one-third is submucosal [8]. In our study, the absence of submucosa was used as an exclusion criterion, and the diagnosis was based on serial sections and immunohistochemical labeling. However, professional recommendations are inconsistent regarding the value of adding IHC to conventional H&E staining [19,41]. The statistically significant difference between nerve fiber thickness quantified by S-100 immunolabeling (*p*) and the presence of calretinin-positive intramucosal nerve fibers in the absence of ganglion cells (<0.001) shows that in small biopsies, both ganglion cells and nerve fibers of the submucosal layer may be obscured by orientation.

Calretinin, a member of the calmodulin superfamily, is a 29-kilodalton, calcium-binding protein [44]; it is highly sensitive and specific for the detection of Meissner and Auerbach plexus ganglion cells (nuclear and cytoplasmic staining are considered positive markers) and is primarily used in the diagnosis of HD [18,45]. It is also expressed in a wide variety of normal and tumor tissues [46] but without expression in other normal cells of the gastrointestinal tract. It is recognized that calretinin is superior to acetylcholinesterase in completing histology, and it is easier to use on formalin-fixed tissue alone or in combination with other markers [47]; as a result, for the histological characterization of the biopsies, we used a 140 kDa isoform of the neural cell adhesion molecule (NCAM) in combination with the CD56 antigen [48,49]. Virtually all axons and ganglion cells in the nerve plexus (Auerbach’s and Meissner’s) show a moderate–strong, distinct, and predominantly membranous staining reaction [50].

Ganglion cells are labeled with calretinin and CD56; as a result, the presence of these cells can be detected even in small biopsies with artifactual changes or partially obscured by surrounding supporting cells. Thus, immunolabeling is further supported by S-100 immunostaining, with prominent negative cells surrounded by positive Schwann cells and nerve fibers. In newborns, where immature ganglion cells (frequently calretinin-negative but CD56-positive) mimic endothelial or even fibroblastic cells [10], the concomitant use of CD56 and calretinin decreases the possibility of a false-positive diagnosis of HD [10,20]. However, S-100 also highlights well-thickened nerve fibers, an evident histologic sign of HD [23], and improves the accuracy of morphometric measurements. The statistically significant disparities in NT sizes, with NT diameters greater than 40 between biopsies and operative specimens (<0.001), underscore the importance of assessing NT diameter in all examined tissue samples.

The main treatment for Hirschsprung is surgery [51]. In this procedure, the aganglionic bowel segment is removed, and the healthy bowel segment is lowered at a point above the dentate line. In some cases, it is necessary to temporarily pass a portion of the colon (colostomy) or small intestine (ileostomy) through the abdominal wall [52]. We described a temporary colostomy–ileostomy in 31.25% of our cases. It is also worth noting that individuals who were first diagnosed during the surgery were admitted to the hospital multiple times. The follow-up of all patients showed a significant difference in re-admission to the hospital between the two groups studied in favor of the surgical group, highlighting the importance of performing rectal biopsies when HD is suspected, with appropriate management to avoid emergency surgery.

Our study results were limited by the fact that a fully paired comparison of the histological picture in biopsies and surgical specimens could not be made because 22 cases did not undergo surgery at our center.

The diagnosis of HD is not the sole responsibility of the pathologist. The management of a child with HD should be conducted by an experienced and multidisciplinary team. A diagnosis of certainty requires biopsy material of adequate quality and quantity for histopathologic examination taken from an area representative of GCs. Such material must be obtained from a region of the patient that is representative of the GCs of interest. Furthermore, the material must undergo complex histological processing, and a correct interpretation of any modifications must be made. Should the pathologist entertain any doubts about the diagnosis, they are obliged to discuss the case with the attending physician.

In this context, we believe that the proposed outcome-based diagnostic algorithm for the diagnosis of HD would facilitate the optimization of the processing and evaluation of intestinal biopsies, thus initiating the stepwise and timely implementation of histological and immunohistochemical methods to confirm or exclude aganglionosis.

## 5. Conclusions

The early diagnosis of HD is important to avoid major complications (e.g., intestinal obstruction, enterocolitis, or toxic megacolon) that negatively affect treatment outcomes. The main signs of the disease should not be underestimated, and in case of clinical and imaging suspicion, biopsy should be performed. Coupled calretinin–CD56 immunohistochemistry is useful for diagnosing ganglion cell paucity biopsies or specimens with incomplete submucosa. In cases where there are no ganglion cells but there are calretinin-positive nerve fibrils in the lamina propria without (NT) hypertrophy, re-biopsy is the best solution. The significant differences in NT size between biopsies and surgical specimens highlight the importance of assessing NT diameter in all tissue samples examined.

## Figures and Tables

**Table 1:** Descriptive statistics of the diagnostic variables in the biopsy specimen group (n = 32).

Variables	Values	*p*-Value
Age (years)	5.7 ± 0.9	
Sex (male/female)	19 (59.4)/13 (40.6)	<0.001
Comorbidities (yes/no)	5 (15.63)/27 (84.37)	0.022
Number of admissions to hospital	Average 3.59	
Number of biopsies (1-2-3-4-5)	7 (21.8)/20 (62.5)/2 (6.2)/2 (6.2)/1 (3.1)	
Rectal biopsy only/other localizations	23 (71.88)/9 (28.12)	
Distance of rectal biopsy from dentate line (cm)	3.1 ± 0.7	-
Calretinin positivity, ganglion cell body (yes/no)	0 (0)/32 (100)	-
Intramucosal calretinin-positive fibers (yes/no)	3 (9.37)/29 (90.63)	<0.001
CD56+/calretinin vs. double-negative ganglion cells	6 (18.75)/26 (81.25)	0.012
Width of fiber (μm)	42.2 ± 2	-
Class of fiber width (<40 µm/>40 µm)	10 (31.25)/22 (68.75)	<0.001

Categorical variables are shown as absolute numbers and percentages (in brackets). Continuous variables are indicated as mean ± SE. *p*-values were calculated with the one-sample *t*-test, and the threshold of significance was set to <0.05.

**Table 2:** Patients who underwent surgery and characteristics of their histological specimens (n = 16).

Variables	Values	*p*
Age (years)	4.3 ± 1.2	
Sex (male/female)	11 (68.7)/5 (31.3)	<0.001
Surgical intervention (temporary colostomy–ileostomy/pull-through)	5 (31.25)/11 (68.75)	-
Postoperative complication, short-term outcomes (yes/no)	4 (25)/12 (75)	<0.001
Postoperative complication, long-term outcomes (yes/no)	12 (75)/4 (25)	
Number of serial biopsies performed at time of surgery	6 ± 0.5	
Width of fiber (μm)	87.6 ± 1.2	
Fiber width class (≤40 µm/>40 µm)	0/16	<0.001
Calretinin-positive fibers (yes/no)	0/0	-
CD56-positive/calretinin-negative ganglion cells vs. double-negative	2 (12.5)/14 (87.5)	<0.001
Type (short segment/long/total form)	4 (25)/11 (68.75)/1 (6.25)	
HD-associated enteritis	12 (75)	-
Intestinal occlusion on admission	13 (81.2)	-
Number of admissions to hospital	Average 5.93	-
Comorbidities (yes/no)	4 (25)/12 (75)	<0.001
Follow-up period (years)	3.8 ± 0.5	-
Outcome (favorable/unfavorable)	13 (81.2)/3 (18.8)	-

Categorical variables are shown as absolute numbers and percentages (in brackets). Continuous variables are indicated as mean ± SE. *p*-values were calculated with the one-sample *t*-test, and the threshold of significance was set to <0.05.

**Table 3:** Hospitalization and long-term complications vary by aganglionosis type and association with other malformations.

Type of Aganglionosis	Total No of Admissions (Average: 5.93)	Long-Term Outcomes (11/68.75%)	Other Malformation (4/25%)
Short-segment type (4)	14 (average: 4)	2/4 (25%)	2/4 (50%)
Long-segment type (11)	68 (average: 6.18)	8/11 (73%)	2/11 (18.2%)
Total form (1)	10	1/1 (100%)	0/1

**Table 4:** Comparative analysis of clinical and histopathologic findings in the two study groups.

Variables	Values		*p*
	Biopsies	Surgical Specimens	
Age (years)	5.7 ± 0.9	4.3 ± 1.2	<0.001
Sex (male/female %)	59.4/40.6	68.8/31.2	0.537
Fiber width (μm)	42.2 ± 2	57.6 ± 1.2	<0.001
Fiber width class (≤40 µm)	68.75%	100%	0.015
Intramucosal calretinin-positive fibers	9.37%	0%	<0.001
Calretinin-negative/CD56-positive ganglion cells	18.75%	12.5%	0.589
Number of hospital admissions (min 1–max 12)	3.59 ± 0.45	5.93 ± 1.15	<0.001

Continuous variables are indicated as mean ± SE. Groups were compared using the Mann–Whitney U test. *p*-values were calculated with the one-sample *t*-test; the threshold of significance was set to <0.05.

## Data Availability

https://data.mendeley.com/drafts/2vt82dmpn7 (accessed on 13 January 2025), doi: 10.17632/2vt82dmpn7.1.

## Supplementary Materials

The following supporting information can be downloaded at https://www.mdpi.com/article/10.3390/life15030329/s1, Figure S1: Possibilities of detecting Meissner’s submucosal plexus ganglion cells in a normal rectal biopsy section by immunohistochemistry

## Abbreviations

The following abbreviations are used in this manuscript:

HD	Hirschsprung’s disease
NT	nerve trunk
ENS	enteric nervous system
INFs	intrinsic nerve fibers
H&E	hematoxylin and eosin
AChE	acetylcholinesterase histochemistry
CB	complete biopsy

## References

[1] Goldstein A.M., Hofstra R.M., Burns A.J. (2013). Building a brain in the gut: Development of the enteric nervous system. Clin. Genet..

[2] Neuvonen M.I., Kyrklund K., Lindahl H.G., Koivusalo A.I., Rintala R.J., Pakarinen M.P. (2015). A population-based, complete follow-up of 146 consecutive patients after transanal mucosectomy for Hirschsprung disease. J. Pediatr. Surg..

[3] Kawaguchi A.L., Guner Y.S., Sømme S., Quesenberry A.C., Arthur L.G., Sola J.E., Downard C.D., Rentea R.M., Valusek P.A., Smith C.A. (2021). American Pediatric Surgical Association Outcomes and Evidence-Based Practice (OEBP) Committee. Management and outcomes for long-segment Hirschsprung disease: A systematic review from the APSA Outcomes and Evidence Based Practice Committee. J. Pediatr. Surg..

[4] Yan J., Sun J., Wu R., Tan S.S., Chen Y., Peng Y., Chen Y. (2020). Barium enema findings in total colonic aganglionosis: A single-center, retrospective study. BMC Pediatr..

[5] Stenström P., Kyrklund K., Bräutigam M., Engstrand Lilja H., Juul Stensrud K., Löf Granström A., Qvist N., Söndergaard Johansson L., Arnbjörnsson E., Borg H. (2020). Total colonic aganglionosis: Multicentre study of surgical treatment and patient-reported outcomes up to adulthood. BJS Open.

[6] Sergi C. (2015). Hirschsprung’s disease: Historical notes and pathological diagnosis on the occasion of the 100(th) anniversary of Dr. Harald Hirschsprung’s death. World J. Clin. Pediatr..

[7] Kyrklund K., Sloots C.E.J., de Blaauw I., Bjørnland K., Rolle U., Cavalieri D., Francalanci P., Fusaro F., Lemli A., Schwarzer N. (2020). ERNICA guidelines for the management of rectosigmoid Hirschsprung’s disease. Orphanet J. Rare Dis..

[8] Vervloet G., De Backer A., Heyman S., Leyman P., Van Cauwenberge S., Vanderlinden K., Vercauteren C., Vervloessem D., Miserez M. (2023). Rectal Biopsy for Hirschsprung’s Disease: A Multicentre Study Involving Biopsy Technique, Pathology and Complications. Children.

[9] Ambartsumyan L., Smith C., Kapur R.P. (2020). Diagnosis of Hirschsprung Disease. Pediatr. Dev. Pathol..

[10] Szylberg L., Marszałek A. (2014). Diagnosis of Hirschsprung’s disease with particular emphasis on histopathology. A systematic review of current literature. Prz. Gastroenterol..

[11] Das K., Mohanty S. (2017). Hirschsprung Disease—Current Diagnosis and Management. Indian. J. Pediatr..

[12] Lotfollahzadeh S., Taherian M., Anand S. (2023). Hirschsprung Disease. StatPearls [Internet].

[13] Agrawal R.K., Kakkar N., Vasishta R.K., Kumari V., Samujh R., Rao K.L. (2015). Acetylcholinesterase histochemistry (AChE)—A helpful technique in the diagnosis and in aiding the operative procedures of Hirschsprung disease. Diagn. Pathol..

[14] Badizadegan K., Thomas A.R., Nagy N., Ndishabandi D., Miller S.A., Alessandrini A., Belkind-Gerson J., Goldstein A.M. (2014). Presence of intramucosal neuroglial cells in normal and aganglionic human colon. Am. J. Physiol. Gastrointest. Liver Physiol..

[15] Zemheri E., Engin Zerk P., Ulukaya Durakbasa C. (2021). Calretinin immunohistochemical staining in Hirschsprung’s disease: An institutional experience. North. Clin. Istanb..

[16] de Arruda Lourenção P.L., Takegawa B.K., Ortolan E.V., Terra S.A., Rodrigues M.A. (2013). A useful panel for the diagnosis of Hirschsprung disease in rectal biopsies: Calretinin immunostaining and acetylcholinesterase histochesmistry. Ann. Diagn. Pathol..

[17] Yoshimaru K., Yanagi Y., Obata S., Takahashi Y., Irie K., Omori A., Matsuura T., Taguchi T. (2021). Acetylcholinesterase staining for the pathological diagnosis of Hirschsprung’s disease. Surg. Today.

[18] Alexandrescu S., Rosenberg H., Tatevian N. (2013). Role of calretinin immunohistochemical stain in evaluation of Hirschsprung disease: An institutional experience. Int. J. Clin. Exp. Pathol..

[19] Galazka P., Szylberg L., Bodnar M., Styczynski J., Marszalek A. (2020). Diagnostic Algorithm in Hirschsprung’s Disease: Focus on Immunohistochemistry Markers. In Vivo.

[20] Holland S.K., Hessler R.B., Reid-Nicholson M.D., Ramalingam P., Lee J.R. (2010). Utilization of peripherin and S-100 immunohistochemistry in the diagnosis of Hirschsprung disease. Mod. Pathol..

[21] Smith C., Ambartsumyan L., Kapur R.P. (2020). Surgery, Surgical Pathology, and Postoperative Management of Patients With Hirschsprung Disease. Pediatr. Dev. Pathol..

[22] Moore E.I., Baxendene J.J., Lowe D.G., Underwood J.C.E. (2001). Diagnosis and molecular pathology of Hirschsprung’s disease. Recent Advances in Histopathology.

[23] Conces M.R., Beach S., Pierson C.R., Prasad V. (2022). Submucosal Nerve Diameter in the Rectum Increases With Age: An Important Consideration for the Diagnosis of Hirschsprung Disease. Pediatr. Dev. Pathol..

[24] Patandianan Y.T., Nurmantu F., Mariana N., Miskad U.A., Zainuddin Ahmadwirawan Sulmiati A.A., Rubiyanto Habar T., Kusuma Prihantono M.I., Faruk M. (2021). Relationship of nerve diameter using S-100 immunohistochemistry with Hirschsprung-associated enterocolitis degrees. Med. Clín. Práct..

[25] Kaji T., Yamada W., Baba T., Machigashira S., Taguchi T., Matsufuji H., Ieiri S. (2019). Classification. Hirschsprung’s Disease and the Allied Disorders.

[26] Khan A.R., Vujanic G.M., Huddart S. (2003). The constipated child: How likely is Hirschsprung’s disease?. Pediatr. Surg. Int..

[27] Rahman Z., Hannan J., Islam S. (2010). Hirschsprung’s disease: Role of rectal suction biopsy—Data on 216 specimens. J. Indian. Assoc. Pediatr. Surg..

[28] Croffie J.M., Davis M.M., Faught P.R., Corkins M.R., Gupta S.K., Pfefferkorn M.D., Molleston J.P., Fitzgerald J.F. (2007). At what age is a suction rectal biopsy less likely to provide adequate tissue for identification of ganglion cells?. J. Pediatr. Gastroenterol. Nutr..

[29] Best K.E., Addor M.C., Arriola L., Balku E., Barisic I., Bianchi F., Calzolari E., Curran R., Doray B., Draper E. (2014). Hirschsprung’s disease prevalence in Europe: A register based study. Birth Defects Res. A Clin. Mol. Teratol..

[30] Amiel J., Sproat-Emison E., Garcia-Barcelo M., Lantieri F., Burzynski G., Borrego S., Pelet A., Arnold S., Miao X., Griseri P. (2008). Hirschsprung Disease Consortium. Hirschsprung disease, associated syndromes and genetics: A review. J. Med. Genet..

[31] Amiel J., Lyonnet S. (2001). Hirschsprung disease, associated syndromes, and genetics: A review. J. Med. Genet..

[32] Pini Prato A., Rossi V., Mosconi M., Holm C., Lantieri F., Griseri P., Ceccherini I., Mavilio D., Jasonni V., Tuo G. (2013). A prospective observational study of associated anomalies in Hirschsprung’s disease. Orphanet J. Rare Dis..

[33] Karim A., Tang C.S., Tam P.K. (2021). The Emerging Genetic Landscape of Hirschsprung Disease and Its Potential Clinical Applications. Front. Pediatr..

[34] Anderson J.E., Vanover M.A., Saadai P., Stark R.A., Stephenson J.T., Hirose S. (2018). Epidemiology of Hirschsprung disease in California from 1995 to 2013. Pediatr. Surg. Int..

[35] Granéli C., Dahlin E., Börjesson A., Arnbjörnsson E., Stenström P. (2017). Diagnosis, Symptoms, and Outcomes of Hirschsprung’s Disease from the Perspective of Gender. Surg. Res. Pract..

[36] Ziad F., Katchy K.C., Al Ramadan S., Alexander S., Kumar S. (2006). Clinicopathological features in 102 cases of Hirschsprung disease. Ann. Saudi Med..

[37] Ho J.M.D., How C.H. (2020). Chronic constipation in infants and children. Singap. Med. J..

[38] Munnangi P., Sayed Mushir Ali A., Deva S., Kushwaha V., Srivastava S., Boini A., Agarwal R.S., Dinkar P.K., Chaudhary E. (2023). Post-surgical Outcomes of Different Surgical Techniques in Hirschsprung’s Disease: A Literature Review. Cureus.

[39] Serafini S., Santos M.M., Tannuri A.C.A., Di Loreto C., Gonçalves J.O., Tannuri U. (2023). A new systematization of histological analysis for the diagnosis of Hirschsprung’s disease. Clinics.

[40] Keyzer-Dekker C.M., Sloots C.E., Schokker-van Linschoten I.K., Biermann K., Meeussen C., Doukas M. (2016). Effectiveness of Rectal Suction Biopsy in Diagnosing Hirschsprung Disease. Eur. J. Pediatr. Surg..

[41] Korsager L.E.H., Bjørn N., Ellebæk M.B., Christensen L.G., Qvist N. (2023). Full-Thickness Rectal Biopsy in Children Suspected of Having Hirschsprung’s Disease: The Inconclusive Biopsy. Children.

[42] Muise E.D., Cowles R.A. (2016). Rectal biopsy for Hirschsprung’s disease: A review of techniques, pathology, and complications. World J. Pediatr..

[43] Schwaller B. (2014). Calretinin: From a “simple” Ca(2+) buffer to a multifunctional protein implicated in many biological processes. Front. Neuroanat..

[44] Alturkustani M., Shillingford N., Zhou S., Wang L., Warren M. (2021). Phox2b Immunohistochemical Staining in Detecting Enteric Neural Crest Cells in Hirschsprung Disease. Pediatr. Dev. Pathol..

[45] Lugli A., Forster Y., Haas P., Nocito A., Bucher C., Bissig H., Mirlacher M., Storz M., Mihatsch M.J., Sauter G. (2003). Calretinin expression in human normal and neoplastic tissues: A tissue microarray analysis on 5233 tissue samples. Hum. Pathol..

[46] Guinard-Samuel V., Bonnard A., De Lagausie P., Philippe-Chomette P., Alberti C., El Ghoneimi A., Peuchmaur M., Berrebi-Binczak D. (2009). Calretinin immunohistochemistry: A simple and efficient tool to diagnose Hirschsprung disease. Mod. Pathol..

[47] Van Acker H.H., Capsomidis A., Smits E.L., Van Tendeloo V.F. (2017). CD56 in the Immune System: More Than a Marker for Cytotoxicity?. Front. Immunol..

[48] Shimamoto M., Ueno Y., Tanaka S., Onitake T., Hanaoka R., Yoshioka K., Hatakeyama T., Chayama K. (2007). Selective decrease in colonic CD56(+) T and CD161(+) T cells in the inflamed mucosa of patients with ulcerative colitis. World J. Gastroenterol..

[49] https://www.pathologyoutlines.com/topic/cdmarkerscd56.html.

[50] Green H.L., Rizzolo D., Austin M. (2016). Surgical management for Hirschsprung disease: A review for primary care providers. JAAPA.

[51] Widyasari A., Pavitasari W.A., Dwihantoro A., Gunadi (2021). Correction to: Functional outcomes in Hirschsprung disease patients after transabdominal Soave and Duhamel procedures. BMC Gastroenterol..

[52] Hutchings E.E., Townley O.G., Lindley R.M., Murthi G.V.S. (2023). The role of stomas in the initial and long-term management of Hirschsprung disease. J. Pediatr. Surg..

