# Supplementary material for: Histopathologic Concerns and Diagnostic Challenges in Hirschsprung’s Disease: An Eastern European Single-Center Observational Study
Source: Life (Basel). 2025 Feb 20;15(3):329. doi: 10.3390/life15030329 (PMC11943527; doi:10.3390/life15030329)
Supplement: Supplementary file 1 [file life-15-00329-s001.zip › Figure S1.pdf]

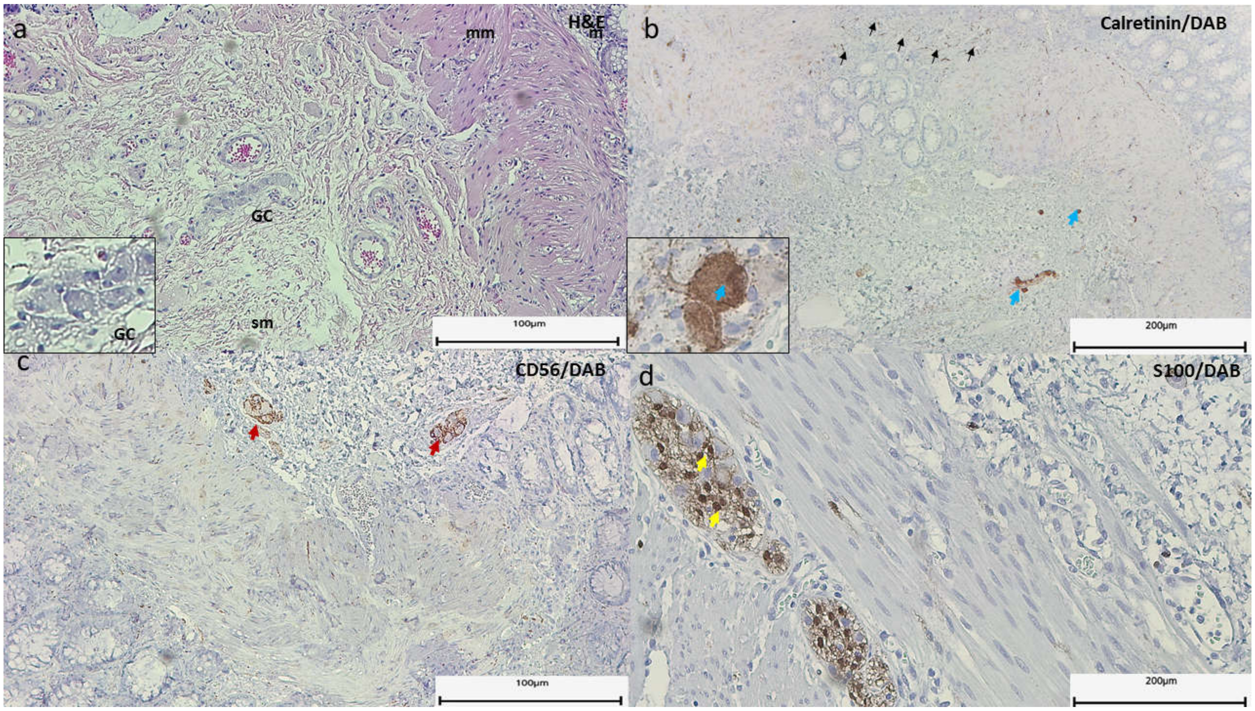

Figure S1. Possibilities of detecting Meissner's submucosal plexus ganglion cells in a normal rectal biopsy section by immunohistochemistry. a. Normal rectal biopsy section (Control group-H&E staining, scale bar 100 µm). The sm shows GCs with vesicular nuclei and prominent nucleoli surrounded by abundant cytoplasm (Inset). (m-mucosa, mm-muscularis mucosae, sm-submucosa, GC-ganglion cell). b. Calretinin/DAB immunohistochemistry highlights positive GCs (light blue arrows) and thin linear granular intrinsic nerve fibres extending in the lamina propria (black arrows), scale bar 200 µm. Inset: Calretinin/DAB-positive GCs with nuclear and cytoplasmic staining surrounded by negative Schwann cells (x400 magnification). c. In the CD56/DAB reaction, GCs are visualised by the continuous and strong membrane reaction (red arrows), scale bar 100 µm. d. The presence of GCs is supported by S-100 immunostaining as prominent negative cells surrounded by positive Schwann cells (yellow arrows), scale bar 200 µm.
